# Supplementary material for: Association between lignan polyphenol bioavailability and enterotypes of isoflavone metabolism: A cross-sectional analysis
Source: PLoS One. 2023 Dec 4;18(12):e0295185. doi: 10.1371/journal.pone.0295185 (PMC10695372; doi:10.1371/journal.pone.0295185)
Supplement: S1 Table — (DOCX) [file pone.0295185.s001.docx]

**Supplemental Table S1. The ratio of EQP enterotype to O-DMAP enterotype.**

|  |  | **EQP status** |  |  |  |  |
| --- | --- | --- | --- | --- | --- | --- |
|  |  | **(+)** | **(-)** | **Odds ratio**  **(95% CI)** | **χ^2^** | **P** |
| **O-DMAP status** | **(+)** | 189 | 164 |  | 15.0 | **<0.0001** |
|  | **(-)** | 28 | 63 | 2.59  (1.58 - 4.24) |  |  |

**Legend for S1 Table:**

O-DMAP: O-DMA producer; EQP: equol producer; χ^2^; result from chi-square test.

P values indicate the results from Pearson's chi-square test.
