# Supplementary material for: Association between lignan polyphenol bioavailability and enterotypes of isoflavone metabolism: A cross-sectional analysis
Source: PLoS One. 2023 Dec 4;18(12):e0295185. doi: 10.1371/journal.pone.0295185 (PMC10695372; doi:10.1371/journal.pone.0295185)
Supplement: S2 Table — (DOCX) [file pone.0295185.s002.docx]

**Supplemental Table S2. Pearson’s correlation between urinary log DAI values and urinary log ENL and log END values by EQP enterotype and O-DMAP enterotype.**

| Enterotypes | **N** | ***r* for log END (μmol/g-Cr)** | **P** | **r for log ENL (μmol/g-Cr)** | **P** |
| --- | --- | --- | --- | --- | --- |
| log DAI (μmol/g-Cr) |  |  |  |  |  |
| O-DMAP/EQP | 189 | 0.244 | 0.000704 | 0.254 | 0.000427 |
| O-DMAP/Non EQP | 164 | 0.0544 | 0.489 | 0.103 | 0.191 |
| Non O-DMAP/EQP | 28 | -0.306 | 0.113 | -0.0365 | 0.854 |
| Non O-DMAP/Non EQP | 63 | 0.000968 | 0.994 | 0.0193 | 0.881 |

**Legend for S2 Table:**

END: enterodiol; ENL: enterolactone; Log: common logarithm; g-Cr: grams creatinine; DAI: daidzein; O-DMAP: O-DMA producer; EQP: equol producer.
