# Supplementary material for: Association between lignan polyphenol bioavailability and enterotypes of isoflavone metabolism: A cross-sectional analysis
Source: PLoS One. 2023 Dec 4;18(12):e0295185. doi: 10.1371/journal.pone.0295185 (PMC10695372; doi:10.1371/journal.pone.0295185)
Supplement: Attachment — Submitted filename: Reviewer1016.docx [file pone.0295185.s003.docx]

**Responses for reviewers’ comments**

**Dear Editor and reviewers,**

**Thank you for valuable feedbacks to our manuscript. We revised our manuscript based on your suggestions. Please see following responses and revisions.**

**In addition, according to the editorial instructions, ethical statements were moved from Acknowledgments section to Methods section (L74–78, L298–301).**

**Data availability section was revised to state the reason for the restriction (L306–309).**

Reviewer #1: The manuscript aims at studying of lignan metabolism using modern analytical methods. I have the following questions, comments and remarks:

Comments and suggestions:

#1. Abstract. The experimental groups should be defined.

**(response)**

**Thank you for your comments.**

**We revised the manuscripts as suggested to include the definition of EQP and O-DMAP (L18–20 in changes-tracked manuscript).**

*EQP was defined by urinary daidzein (DAI) and equol concentrations as log(equol/DAI) ≥   –1.42. O-DMAP was defined by urinary DAI and O-DMA concentrations as O-DMA/DAI > 0.018.*

#2. Experimental procedures/ Results and Discussion. Did the authors monitor the changes of the detected compounds in time, i.e. the time of storage?

**(response)**

**Thank you for the valuable comment.**

**Changes over time of the detected compounds were not measured in this study. But in our previous study, we evaluated the stability of isoflavones and lignans. We revised the manuscripts to include this information (L107–109 in changes-tracked manuscript).**

*Changes over time in the target compounds during storage were not evaluated in this study, but in our previous study [17], the archived samples used from the same specimen bank showed similar average concentrations of isoflavones and lignans to fresh samples.*

#3. Conclusion

I would suggest to response minor issues prior to further consideration of the work. Based on my concerns, minor revision is required.

Thank you for your evaluation and kind comments.

Reviewer #2: I have reviewed the manuscript titled Lignan polyphenol metabolism is partially determined by isoflavone metabolism enterotypes:

a cross-sectional analysis, and I appreciate the authors' efforts in conducting this research. However, after careful evaluation, I regret to inform you that I cannot recommend this manuscript for publication in its current form due to “Lack of Significance”, “Weak Methodology”, “Less Clarity in Data Presentation”“Less Statistical　Significance”.

I encourage the authors to consider revising the manuscript, addressing the aforementioned concerns, and potentially exploring alternative avenues for publication.

**(response)**

**Thank you for your comments. We revised the manuscript regarding for “Lack of Significance”, “Weak Methodology”,“Less Clarity in Data Presentation”, “Less Statistical Significance”.**

**“Lack of Significance”**

**(response)**

**We emphasized the significance of the study more in the discussion section. (L231–233 in changes-tracked manuscript)**

*There have not been reported for determinants of availability of lignan polyphenols while inter-individual variability was found. In the present study, we examined individual differences in urinary END and ENL concentrations for enterotypes of isoflavone metabolisms.*

**“Weak Methodology”**

**(response)**

**We have already written the limitation of the study in the Discussion section, but we also added it to the abstract as well. (L28–31 in changes-tracked manuscript)**

*The variables and participants in this study was limited, which the possibility of confounding by other variables cannot be ruled out. However, there are no established determinants of lignan metabolism to date. Further research is needed to determine what factors should be considered, and to examine in different settings to confirm the external validity.*

**“Less Clarity in Data Presentation”**

**(response)**

**Since** **the primary outcome is the relationship between gut microbiota types and urinary lignans, this is clearly indicated in the tables and figures. But we now clearly indicate it in the statistical analysis section (L112 in changes-tracked manuscript).**

*The primary outcome is the relationship between four enterotypes and urinary lignans concentrations.*

**“Less Statistical　Significance”.**

**(response)**

**The analysis of the primary outcome shows sufficient statistical significances (P<0.01 to P<0.001).**

**The p-values have been added to the Abstract (L24–26)**

*The urinary lignan concentration was significantly higher in the O-DMAP/EQP group (ENL: P<0.001, END: P<0.001), and this association remained significant after adjusting for several background variables (END:β=0.138 P=0.00607 for EQP, β=0.147 P= 0.00328 for O-DMAP; ENL:β=0.312, P<0.001 for EQP, β=0.210, P<0.001 for O-DMAP).*
