# Supplementary material for: Association between lignan polyphenol bioavailability and enterotypes of isoflavone metabolism: A cross-sectional analysis
Source: PLoS One. 2023 Dec 4;18(12):e0295185. doi: 10.1371/journal.pone.0295185 (PMC10695372; doi:10.1371/journal.pone.0295185)
Supplement: Attachment — Submitted filename: responses20231115.docx [file pone.0295185.s004.docx]

**Responses for comments**

**Dear Editor and Reviewer,**

**Thank you for valuable feedbacks to our manuscript. We revised our manuscript based on your suggestions. Please see following responses and revisions.**

**Editor’s comment**

Thank you for submitting your value work in PLOS ONE, your positive response is appreciated.
I agree with reviewer2 that the title need improving and I am suggesting "A cross-sectional analysis of Lignan polyphenol partially determination by isoflavone metabolism enterotypes", or you can improve it by yourself, the word metabolism is written twice, and this may confuse the readers.

**(response)**

**Thank you for your comment.**

**As the Editor and Reviewer 2 suggested, we revised the title to “Association between lignan polyphenol bioavailability and enterotypes of isoflavone metabolism: a cross-sectional analysis” (L1–2 in changes-tracked manuscript).**

**In addition, we reviewed the manuscript and edits for typos.**

**Reviewer #1:** The manuscript was corrected according to reviewers comments. Therefore, the manuscript may be accepted.

**(response)**

We appreciate your efforts in this review and evaluation of our study.

**Reviewer #2:**

Dear Authors, You've made good efforts to address the comments. I recommend a minor revision:

#1: Make the title more catchy and concise. For instance, consider "Cross-Sectional Analysis of Lignan Polyphenol Metabolism and Isoflavone Enterotypes."

**(response)**

**Thank you for your comment.**

**As the Editor and Reviewer 2 suggested, we revised the title to “Association between lignan polyphenol bioavailability and enterotypes of isoflavone metabolism: a cross-sectional analysis” (L1–2 in changes-tracked manuscript).**

#2: Ensure the conclusion is clear by summarizing the main findings, discussing their significance, addressing research questions and limitations, and providing data-based recommendations for future research.

**(response)**

**Thank you for the suggestion.**

**We revised the Conclusion section as suggested (L287–294 in the changes-tracked manuscript).**

*In this study, the factors that are responsible for individual differences in the bioavailability of ENL and END were investigated in relation to enterotypes of isoflavone metabolism. Urinary END and ENL concentrations were 3.80- and 10.2-fold higher in the EQP/O-DMAP group than in the non-EQP/non-O-DMAP group, respectively. However urinary isoflavone concentrations were not associated with END and ENL concentrations. Dietary intake of lignan polyphenols may not represent precise bioavailable amounts and these enterotypes may provide better estimates. The possibility of biases and confounding cannot be ruled out due to the limitations in observed variables and participants. Further research to determine causative gut bacteria is needed.*

**Also, the values of lignans are provided (L156–160).**

*The geometric mean of the urinary log END (0.0473 μmol/g-Cr) value in non-O-DMAP/non-EQP was lower than for O-DMAP/EQP and O-DMAP/non-EQP (0.180 and 0.119 μmol/g-Cr; P < 0.001 and P = 0.0025, respectively). Also, urinary log ENL (0.0176 μmol/g-Cr) concentrations were lower in non-EQP/non-O-DMAP than in EQP/O-DMAP, non-EQP/O-DMAP, and non-O-DMAP/EQP (0.181, 0.0577 and 0.0943 μmol/g-Cr; P < 0.001, P < 0.001, and P = 0.0003, respectively).*
